# Determination of SGK1 mRNA in non-small cell lung cancer samples underlines high expression in squamous cell carcinomas

**DOI:** 10.1186/1756-9966-31-4

**Published:** 2012-01-12

**Authors:** Claudia Abbruzzese, Stefano Mattarocci, Laura Pizzuti, Anna M Mileo, Paolo Visca, Barbara Antoniani, Gabriele Alessandrini, Francesco Facciolo, Rosario Amato, Lucia D'Antona, Massimo Rinaldi, Armando Felsani, Nicola Perrotti, Marco G Paggi

**Affiliations:** 1 Department of Development of Therapeutic Programs, National Cancer Institute "Regina Elena", IRCCS, 00144 Rome, Italy; 2 Medical Oncology Division B, National Cancer Institute "Regina Elena", Via Elio Chianesi, 53, 00144 Rome, Italy; 3 Department of Pathology, Regina Elena Cancer Institute, Via E. Chianesi, 53, 00144 Rome, Italy; 4 Department of Oncologic Thoracic Surgery, Regina Elena Cancer Institute, Via E. Chianesi, 53, 00144 Rome, Italy; 5 Department of Experimental and clinical Medicine "G. Salvatore", Faculty of Medicine, University Magna Graecia, 88100 Catanzaro, Italy; 6 CNR, Istituto di Neurobiologia e Medicina Molecolare, Via Fosso di Fiorano, 64, 00143 Rome, Italy

**Keywords:** SGK1, NSCLC, mRNA, quantitative PCR, archival samples, retrospective analysis

## Abstract

**Background:**

Lung cancer represents the most frequent cause of death for cancer. In non-small cell lung cancer (NSCLC), which accounts for the vast majority of this disease, only early detection and treatment, when possible, may significantly affect patient's prognosis. An important role in NSCLC malignancy is attributed to the signal transduction pathways involving PI3Kinase, with consequent activation of the AKT family factors. The serum and glucocorticoid kinase (SGK) factors, which share high structural and functional homologies with the AKT factors, are a family of ubiquitously expressed serine/threonine kinases under the control of cellular stress and hormones. SGK1 is the most represented SGK member.

**Methods:**

By means of immunohistochemistry and quantitative real-time PCR, we determined SGK1 protein and mRNA expression in a cohort of 66 formalin-fixed, paraffin-embedded NSCLC surgical samples. All samples belonged to patients with a well-documented clinical history.

**Results:**

mRNA expression was significantly higher in squamous cell carcinomas, and correlated with several clinical prognostic indicators, being elevated in high-grade tumors and in tumors with bigger size and worse clinical stage. No correlation was found between SGK1 protein expression and these clinical parameters.

**Conclusions:**

This explorative analysis of SGK1 expression in NSCLC samples highlights the potential role of this factor in NSCLC patients' prognosis. Moreover, the higher expression in the squamous cell carcinoma subtype opens new therapeutic possibilities in this NSCLC subtype by designing specific kinase inhibitors.

## Background

Lung cancer represents the foremost cause of cancer death, at least in Western countries [1-3]. From a clinical point of view, lung cancer is classified as "small cell lung cancer" (SCLC) and "non-small cell lung cancer" (NSCLC), the form by far most frequent (about 85% of the total cases). NSCLCs are histopathologically subdivided into adenocarcinoma, squamous cell carcinoma and large cell carcinoma [1]. Recently, this NSCLC subclassification has been shown to reflect also specific epidemiological as well as biological behaviors, which can be epitomized in a higher incidence in never-smokers and in women of the adenocarcinomatous subtype [4-7] and in its higher sensitivity to EGFR tyrosine kinase inhibitors [8].

In NSCLC, a major role is attributed to the membrane-bound tyrosine kinase receptors, mainly EGFR, which in their active, phosphorylated form generate a cascade of biological effects which strongly favor several biological processes, as cell proliferation, neo-angiogenesis and invasive capability [9]. Interestingly, also insulin and insulin receptor have been recently involved in lung epithelial cells transformation [10,11]. A pivotal step of the cascade triggered by tyrosine kinase receptors is the activation of the phosphoinositide-3-kinase (PI3Kinase) pathway, which allows the convergence of several signals in activating the AKT family of serine/threonine kinases, thus stimulating cell growth, mitosis, survival and energy metabolism [12-14]. The serum and glucocorticoid kinase (SGK) family of serine/threonine kinases share many structural and functional similarities with the AKT family, since the metabolic pathways over which both families exert their activity are extremely similar [15]. SGK family is composed of three members, SGK1, SGK2 and SGK3, coded by three different genes, which are in turn subdivided into different splicing variants [16]. SGK1, the most represented member of the SGK family, is ubiquitously expressed and is under the control of cellular stress (including cell shrinkage) and hormones (including gluco-and mineral-corticoids). All isoforms are activated by insulin and other growth factors [15].

SGKs are involved in numerous pathophysiological functions, and, among these, also neoplastic growth, where SGK factors show often enhanced activity, influencing several control mechanisms as cell growth and proliferation [15], cell survival [17,18], cell migration and invasion [19,20].

Recently, our group described the role of insulin and insulin receptor in the early carcinogenic steps of some NSCLCs [11]. Here we used quantitative real-time PCR (qPCR) and immunohistochemistry (IHC) to determine respectively mRNA and protein expression of SGK1 (total and phosphorylated/activated), the most represented family member, in archival NSCLC samples from patients with a well-documented clinical history. This is a retrospective study aiming at characterizing the role of SGK1 in NSCLC onset and progression, and in setting the ground for the possible use of SGK1 as a prognostic factor or therapeutic target.

## Methods

### Patients

Tissues from 66 NSCLC surgical specimens (35 adenocarcinomas, 25 squamous cell carcinomas, plus 6 specimens classified as "other", which are 1 adenosquamous carcinoma, 4 undifferentiated carcinomas and 1 large cell carcinoma) were evaluated. All the patients were diagnosed and treated at the Regina Elena Cancer Institute, Rome, Italy. Patients underwent international standard radio- and/or chemotherapeutic protocols. Clinical data (patient history, diagnosis, staging and survival) were obtained from the National Cancer Institute "Regina Elena" databases. Survival data were integrated by periodic interviews with patients and/or their relatives. Samples were collected according to institutional ethical guidelines. Written informed consent was obtained from the patients for publication of this case report and accompanying images. A copy of the written consent is available for review by the Editor-in-Chief of this journal.

### RNA extraction and Quantitative gene expression analysis in NSCLC archival samples

Total RNA extraction from formalin-fixed, paraffin-embedded (FFPE) NSCLC specimens was done essentially according to the method described in previous papers [21,22], using modifications concerning slice thickness (7.5 μm instead of 10 μm) and optimizing the time for proteinase digestion (5 h). Total RNA extracted was examined and quantified using the 2100 bioanalizer (Agilent, Santa Clara, CA). For qPCR reaction, the 7900 HT thermal cycler (Applied Biosystems, Branchburg, NJ) apparatus was employed, as described previously [11], using the sequence specific primer pairs described in Table 1 [specific for SGK1 (all four isoforms), for each of the four isoforms and for glyceraldehyde-3-phosphate dehydrogenase (GAPDH), a qualitative and quantitative transcripts control].

**Table 1:** Sequences of the primers used for qPCR of transcripts coding for SGK1 (all four isoforms), for each of the four isoforms and for glyceraldehyde-3-phosphate dehydrogenase (GAPDH).

*Gene Symbol*	*Accession Number*	*Sense Primer*	*Antisense Primer *
SGK1 (all 4 isoforms)	N/A	AGGGCAGTTTTGGAAAGGTT	CTGTAAAACTTTGACTGCATAGAACA
SGK1 (isoform 1)	NM_005627.3	GGCACCCTCACTTACTCCAG	GGCAATCTTCTGAATAAAGTCGTT
SGK1 (isoform 2)	NM_001143676.1	CGGTGGAAAATGGTAAACAAA	CTTGATCCACCTTCGTACCC
SGK1 (isoform 3)	NM_001143677.1	GAAGCTATAAAACCCCCTTTGAA	GGCAATCTTCTGAATAAAGTCGTT
SGK1 (isoform 4)	NM_001143678.1	CTTCCTGCTGAGCGGACT	GGCAATCTTCTGAATAAAGTCGTT
GAPDH	NM_002046	AGCCACATCGCTCAGACA	GCCCAATACGACCAAATCC

### Histological examination and IHC

The histological diagnosis was re-evaluated in 2 μm FFPE sections after routine laboratory haematoxylin/eosin staining.

IHC analysis was done as described [11], omitting the antigen retrieval step, and using a primary monoclonal antibody for SGK1 (sc-28338, Santa Cruz Biotechnology, Inc. Santa Cruz, CA), applied overnight (O.N.) at 4°C at a dilution of 1:300. Phospho-SGK1 (pSGK1 Ser422) was detected by means of a rabbit polyclonal antibody (sc-16745, Santa Cruz Biotechnology) applied for 2 h at 4°C at a dilution of 1:100). For both antibodies, optimal working dilution was defined on the basis of titration experiments. The secondary antibody solution and streptavidin-biotin, both contained in the QP900-9L kit (BioGenex, San Ramon, CA.), were applied according to the manufacturer's instructions. Finally, 3-amino-9-ethylcarbazide (AEC substrate kit, ScyTek, Logan, UT) was used as chromogen. Mayer's haematoxylin was used for the nuclear counterstaining. Negative controls for each tissue section were prepared by omitting the primary antibody.

### Scoring and quantification of mRNA expression and immunoreactivity

#### mRNA expression

Progression of the qPCR reaction, performed using the primer pairs specified in Table 1, was monitored. All the experiments were performed in quadruplicate.

#### Immunoreactivity

Two examiners (P.V. and M.G.P.) evaluated independently the staining pattern of SGK1 and phospho-SGK1, with subsequent discussion for the cases in which divergent diagnoses were given. According to the amount of staining, cases were classified in tertiles as follows: a) negative/low; b) medium; c) high.

### Statistical analysis

For quantitative variables, average values were determined, and the non-parametric Mann-Whitney U-test was applied to evaluate statistical significance. All categorical variables were tested for statistical significance by using Pearson's χ^2 ^test or Fisher's exact test. Overall survival (OS) and disease-free survival (DFS) curves were done using the Kaplan-Meier method; the log-rank (Mantel-Cox) test was used to compare survival times between patient groups.

For all statistical tests, a two-tailed *P*-value < 0.05 was considered as statistically significant.

## Results

### SGK1 and phospho-SGK1 protein detection in NSCLC samples

**Figure 1:**
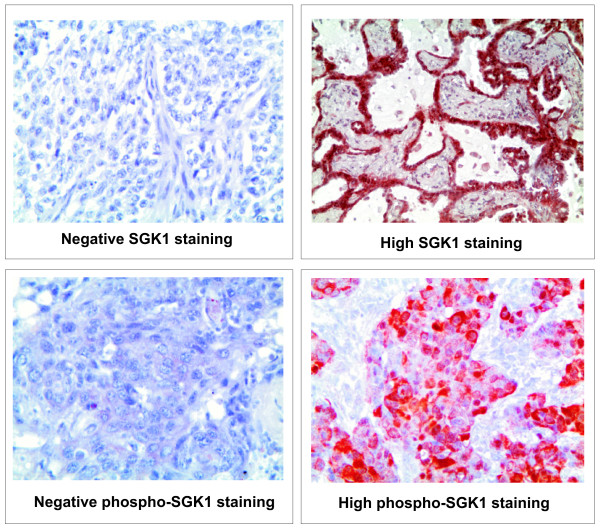
**Immunohistochemical staining for SGK1 and phospho-SGK1**. Representative samples showing negative and high SGK1 staining (sum of all variants) and negative and high phospho-SGK1 in NSCLC. Original magnification = x20.

SGK1 and phospho-SGK1 protein detection was done by IHC on tissue sections from 66 NSCLC specimens from patients with a well-documented clinical history. The antibodies employed did not allow discriminating among the SGK1 forms deriving from the four splicing variants. Samples stained for SGK1 displayed a granular cytoplasmic staining, considered specific due to its absence in the negative controls. Staining appeared non-homogeneous, with an intensity which was variable in different areas of the sample. Samples stained for phospho-SGK1 displayed a granular cytoplasmic staining as well, with a range of intensity comparable to that of SGK1. Figure 1 shows examples of negative and high SGK1 and phospho-SGK1 staining in NSCLC samples. According to staining intensity, samples were subdivided into tertiles, consistent with the scoring given by two pathologists, with null/low, medium and high SGK1 expression. Statistical evaluation found no correlation between SGK1 or phospho-SGK1 staining and the following clinical parameters: a) age at diagnosis; b) gender; c) smoking habit; d) histolopathogical subtype; e) histopathological grade; f) tumor size; g) lymph node stage; h) clinical tumor stage.

### SGK1 mRNA detection in NSCLC samples

By means of the specific primers illustrated in Table 1, we determined the mRNA amount of SGK1 either as the sum of the four different splicing variants or as the value specific for each single variant. In all cases, GAPDH mRNA expression was used for an internal check of the quality of the FFPE-extracted RNA and for normalization. Total SGK1 mRNA expression data, and the values for each splicing variant, were subdivided in tertiles of 22 patients each. Data were challenged against the clinical parameters described above. As far as it concerns the evaluation of the expression of the sum of the four SGK1 mRNA, statistically significant correlation was found with:

**a) histolopathogical subtype **(*P *= 0.022), with the highest expression in squamous cell carcinomas;

**b) histopathological grade **(*P *= 0.026), with the lowest expression in low-grade tumors (G1) and the highest expression in high-grade tumors (G3);

**c) tumor size **(*P *= 0.013), with lower expression in T1 and higher in T3-T4 tumors.

**d) tumor stage **(*P *= 0.028), where the highest expression was found in patients with worse clinical stage.

In this experimental set, no statistical significance was found between SGK1 total mRNA expression and patient's gender, age, smoking history and lymph node stage. All these data are summarized in Table 2. In addition, no correlation between SGK1 mRNA quantification by qPCR and SGK1 protein (or phosphoprotein) expression by IHC was found.

**Table 2:** Evaluation of SGK1 (all variants) mRNA expression in NSCLC samples by qPCR: correlation with clinico-pathological parameters.

		Null/low SGK1 expression n = 22	Medium SGK1 expression n = 22	High SGK1 expression n = 22	*P-value*
**Patient age (years)^§^**		69.1 ± 1.6	66.3 ± 2.4	65.2 ± 1.8	0.386 (NS)

**Gender**	Male	11	13	15	0.471 (NS)
		
	Female	11	9	7	

**Smoking habit**	Smokers	10	12	11	0.834 (NS)
		
	Non-smokers	12	10	11	

**Histopathological Subtype**	Adenocarcinoma	15	12	8	**0.022**
		
	Squamous cell carcinoma	3	10	12	
		
	Other	4	0	2	

**Histopathological Grade**	G1	5	0	1	**0.026**
		
	G2	8	15	9	
		
	G3	9	7	12	

**Tumor Size**	T 1	9	2	6	**0.013**
		
	T 2	12	15	10	
		
	T 3	1	2	6	
		
	T 4	0	3	0	

**Lymph Node Stage**	N 0	18	14	16	0.315 (NS)
		
	N 1	0	4	2	
		
	N 2	3	3	4	
		
	N/A	1	1	0	

**Tumor Stage**	Stage I a	10	2	5	**0.028**
		
	Stage I b	7	10	6	
		
	Stage II a	1	0	0	
		
	Stage II b	1	2	6	
		
	Stage III a	3	4	5	
		
	Stage III b	0	3	0	

§ Average values; **in bold and underlined **= statistically significant results; N.S. = non-significant.

**Figure 2:**
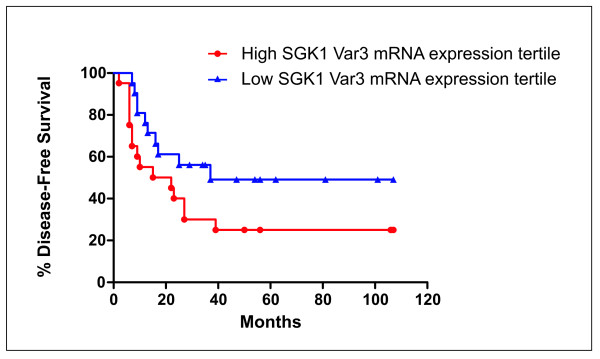
**Disease-Free survival of NSCLC patients with high or low SGK1 variant 3 mRNA expression**. Kaplan-Meier plot representing the disease-free survival of NSCLC patients belonging to the high or low tertile for SGK1 variant 3 mRNA expression. In spite of a discernible trend of better survival for NSCLC patients belonging to the low tertile for SGK1 variant 3 mRNA expression, when compared to the high tertile, with a median value of 37 and 22 mo., respectively, these data are statistically non-significant (*P*-values = 0.083).

When mRNA expression of each single SGK1 splicing variant was considered, lower levels of statistical significance were achieved, as reported below:

1. SGK1 variant 1: significant correlation with histolopathogical subtype (*P *= 0.017), with the highest expression in squamous cell carcinomas; significant correlation with the expression of the sum of the four SGK1 splicing variants (*P *= 4.7 × 10^-6^). Such a high significance was due to the fact that this SGK1 form was by far the most abundant splicing variant;

2. SGK1 variant 2: significant correlation with histolopathogical subtype (p = 0.022), with the highest expression in squamous cell carcinomas; significant correlation with the expression of the sum of the four SGK1 splicing variants (*P *= 0.001);

3. SGK1 variant 3: significant correlation only with the expression of the sum of the four SGK1 splicing variants (*P *= 0.003);

4. SGK1 variant 4: significant correlation only with the expression of the sum of the four SGK1 splicing variants (*P *= 0.008);

When survival data were analyzed (overall survival and disease-free survival), Kaplan-Meier analysis did not reach statistical significance in any cases. The best fitting concerned the expression of SGK1 variant 3 and disease-free survival (*P *= 0.083, non-significant), when only the highest and lowest tertiles were taken into consideration (Figure 2).

## Discussion

The discovery and development of novel predictive tumor biomarkers is a complicated process, and currently the best choice for the identification of reliable markers appears to be an intelligent compromise between the results obtained from high-throughput technologies and the so-called "hypothesis-driven" analyses, which are based upon preliminary selections of factors whose expression is to be estimated (biased approach) [23,24].

Following our previous results on insulin and activated insulin receptor in NSCLC [11], we analyzed in this work the role of SGK1 in NSCLCs by evaluating protein, phosphoprotein and mRNA expression in 66 NSCLC FFPE surgical samples. The data of SGK1 expression showing the best statistical fitting with patients' clinical parameters spring from the mRNA analysis rather than IHC determinations. The most interesting data belong to the set concerning the determination of the mRNA expression of the sum of the four SGK1 splicing variants. Each single splicing variant, when analyzed alone, generated less statistically significant data. From these results, we can assume that the biological role of these different splicing variants goes largely in the same direction, at least in this experimental setting. Essentially, our results showed higher SGK1 transcription in tissue samples from patients with worse clinical prognostic indicators, as, for example, histopathological grading. Among all NSCLC cases, the squamous cell subtype exhibited the highest SGK1 mRNA expression.

Considering SGK1 a factor strongly related to cellular stress, it is not surprising that the highest expression was found in high-grade tumors, because these are usually characterized by higher rates of energy metabolism, which expose them to relative hypo-oxygenation and, paradoxically, to higher oxidative stress due to the Warburg effect [25-28].

A direct correlation between SGK1 protein determination by IHC and tumor malignancy was not found. A possible explanation comes from the notion that the half-life of the four SGK1 protein variants is quite different, being essentially related to the presence or absence of the "ER-motif" in the N-terminal region of the protein, a 6-amino acid sequence responsible for the binding to the endoplasmic reticulum (ER). The ER-motif, when present, imposes a selective localization of the SGK1 molecule on the ER, thus inducing its rapid degradation via the ubiquitin pathway. For this reason, SGK1 variants which possess the ER motif have a half-life by far shorter than the other variants. Indeed, biological activity of SGK1 variants provided of ER motif is mainly regulated via a synthesis/degradation equilibrium [29], while, for the other variants, regulation is mainly due to post-translational modifications (phosphorylation/dephosphorylation) [15].

In this context, it is worth of note that the identification of high SGK1 mRNA expression mainly in the squamous cell subtype of NSCLC may pave the way for specific targeted therapies in this NSCLC subtype. Indeed, currently squamous cell carcinoma appears neglected as far as targeted molecular therapies are considered, being most of these selective molecules employed essentially for the adenocarcinoma subtype. If the role of SGK1 as a specific molecular marker for squamous cell carcinoma will be further validated, an inhibitor of SGK1 kinase activity would be highly appreciated in this NSCLC specific phenotype. Indeed, inhibitors of the AKT family of serine/threonine kinases, structurally and functionally closely related to the SGK factors, have been already described, and their use in clinical trials is underway [30-32].

It seems clear, however, that our knowledge on the role of the SGK family factors in neoplastic diseases is at a very early stage and that further studies are therefore necessary to indicate the most appropriate use of the determination of these kinases in prognostic/predictive evaluation of NSCLC patients as well as the possibility to consider them as a druggable target for specific small molecule inhibitors.

## Conclusions

This work is an explorative study on the role of SGK1, the most represented member of the SGK family of serine/threonine kinases, in NSCLC. The notions derived from our cohort of patients confirm the "oncogenic" role of SGK1, where higher mRNA expression appears related to patients with worse prognostic indicators. Moreover, the significantly higher SGK1 expression in the squamous cell subtype of NSCLC could indicate this factor as central in establishing prognostic/predictive parameters as well as in enforcing the design of SGK serine/threonine kinase inhibitors to be employed in the management of patients with squamous cell lung cancer.

## List of abbreviations

NSCLC: non-small cell lung cancer; SGK1: serum and glucocorticoid-inducible kinase 1; FFPE: formalin-fixed, paraffin-embedded; GAPDH: glyceraldehyde-3-phosphate dehydrogenase; qPCR: quantitative real-time PCR; IHC: immunohistochemistry; ER: endoplasmic reticulum.

## Competing interests

The authors declare that they have no competing interests.

## Authors' contributions

CA: Research planning, IHC and qPCR determinations, statistical analysis. SM: Research planning, IHC and qPCR determinations, statistical analysis. LP: Research planning, collection of patients' information, manuscript drafting. AMM: Research planning and qPCR determinations. PV: Patients' diagnosis, IHC scoring. BA: Tissue slices preparation, haematoxylin/eosin staining. GA: Collection of patients' information, patients' database maintenance. FF: Surgery and patients' database maintenance. RA: qPCR determinations. LD'A: qPCR determinations. MR: Research planning, collection of patients' information, manuscript drafting. AF: Research planning, qPCR determinations, statistical analysis. NP: Research planning, qPCR determinations, statistical analysis, manuscript drafting. MGP: Research planning, coordination of the whole project, IHC scoring, manuscript drafting. All authors read and approved the final manuscript.
